# Donor Heart Preservation with Hydrogen Sulfide: A Systematic Review and Meta-Analysis

**DOI:** 10.3390/ijms22115737

**Published:** 2021-05-27

**Authors:** Imran A. Ertugrul, Vincent van Suylen, Kevin Damman, Marie-Sophie L. Y. de Koning, Harry van Goor, Michiel E. Erasmus

**Affiliations:** 1 University Medical Centre Groningen, Department of Cardiothoracic Surgery, University of Groningen, 9700 RB Groningen, The Netherlands; i.a.ertugrul@umcg.nl (I.A.E.); v.van.suylen@umcg.nl (V.v.S.); m.e.erasmus@umcg.nl (M.E.E.); 2 University Medical Centre Groningen, Department of Cardiology, University of Groningen, 9700 RB Groningen, The Netherlands; k.damman@umcg.nl (K.D.); m.s.l.y.de.koning@umcg.nl (M.-S.L.Y.d.K.); 3 University Medical Centre Groningen, Department of Pathology and Medical Biology, University of Groningen, 9700 RB Groningen, The Netherlands

**Keywords:** hydrogen sulfide, ischemia-reperfusion injury, postconditioning, systematic review, meta-analysis, organ preservation, cardiac transplantation

## Abstract

Preclinical studies have shown that postconditioning with hydrogen sulfide (H_2_S) exerts cardioprotective effects against myocardial ischemia-reperfusion injury (IRI). The aim of this study was to appraise the current evidence of the cardioprotective effects of H_2_S against IRI in order to explore the future implementation of H_2_S in clinical cardiac transplantation. The current literature on H_2_S postconditioning in the setting of global myocardial ischemia was systematically reviewed and analyzed, performing meta-analyses. A literature search of the electronic databases Medline, Embase and Cinahl identified 1835 studies that were subjected to our pre-defined inclusion criteria. Sixteen studies were considered eligible for inclusion. Postconditioning with H_2_S showed significant robust effects with regard to limiting infarct size (standardized mean difference (SMD) = −4.12, 95% CI [−5.53–−2.71], *p* < 0.00001). Furthermore, H_2_S postconditioning consistently resulted in a significantly lower release of cardiac injury markers, lower levels of oxidative stress and improved cardiac function. Postconditioning with slow-releasing H_2_S donors offers a valuable opportunity for novel therapies within cardiac preservation for transplantation. Before clinical implication, studies evaluating the long-term effects of H_2_S treatment and effects of H_2_S treatment in large animal studies are warranted.

## 1. Introduction

Cardiac transplantation is the treatment of choice for patients with end-stage heart failure (HF). The global incidence of end-stage HF is rising, while cardiac transplantation is limited by the severe shortage of donor hearts suitable for transplantation [1]. Donation after brain death (DBD) is the current standard for cardiac transplantation. However, in order to enlarge the donor pool, the use of marginal donors and donation after circulatory death (DCD) has gained interest. One of the prominent challenges in cardiac transplantation is myocardial ischemia-reperfusion injury (IRI) to the graft. IRI is the inevitable result of initial restriction of blood supply to the heart followed by subsequent reperfusion. IRI has not only been associated with microvascular dysfunction, arrhythmias and primary graft dysfunction but also with aggravation of tissue damage that has already occurred by circulatory arrest in marginal donors and DCD hearts [2,3].

Static cold storage (CS) is currently the standard method for minimizing IRI during donor heart preservation. Although CS significantly reduces myocardial metabolism, there is a continued metabolism at a lower level, and ischemic damage occurs over time. Continuation of anaerobic metabolism results in depletion of adenosine triphosphate stores, followed by acidosis, ionic disturbances, production of reactive oxygen species (ROS), oxidative stress and eventually necrosis. Re-establishment of blood flow restores homeostasis and is essential to salvage ischemic tissues. However, reperfusion can paradoxically result in further tissue damage by a quick pH change, activation of inflammatory cascades and cell death programs, calcium (Ca^2+^) overload and increased ROS production [4,5,6]. A maximum period of 4 to 6 h is currently accepted as a safe ischemic preservation time for DBD hearts. Longer ischemic times have been demonstrated to adversely affect post-transplant survival [7]. CS of DCD hearts is insufficient since these hearts have already sustained extensive ischemic injury due to circulatory arrest [8]. Novel strategies to protect the heart from IRI in order to increase the donor pool have been reported, including pharmacological intervention with hydrogen sulfide (H_2_S).

H_2_S is an endogenously produced gasotransmitter generated by cystathionine γ-lyase, cystathionine β-synthase and 3-mercaptopyruvate sulfurtransferase. At high concentrations, gasotransmitters are toxic, but at low concentrations, they function as important signaling molecules [9]. In the cardiovascular system, endogenous H_2_S plays a prominent role in vasodilatation and blood pressure regulation [10]. Multiple exogenous H_2_S-releasing compounds (H_2_S donors) are available and have been extensively researched in diverse pathological processes, including myocardial IRI [11]. An increasing body of evidence indicates that pre- and postconditioning the heart with an exogenous H_2_S donor exerts cardioprotective effects by limiting inflammation and oxidative stress, and by protecting against mitochondrial damage and apoptosis caused by IRI [12,13,14]. In addition, H_2_S is known to act as a regulator of energy production under stress conditions, which sustains mitochondrial ATP production under hypoxic conditions [15]. These beneficial effects of H_2_S, especially in H_2_S postconditioning, could be of additional value in treatment of myocardial infarction (local ischemia) and in the setting of cardiac transplantation and donor heart preservation (global ischemia).

Although H_2_S therapy against IRI has been investigated abundantly in animal studies, no published comprehensive review on postconditioning with H_2_S in the setting of global ischemia is available. The aim of this study was to appraise the current evidence of the cardioprotective effects of H_2_S postconditioning against IRI by systematically reviewing the literature and performing meta-analyses, in order to explore the future implementation of H_2_S in clinical cardiac transplantation. 

## 2. Results

### 2.1. Study Selection Process

**Figure 1:**
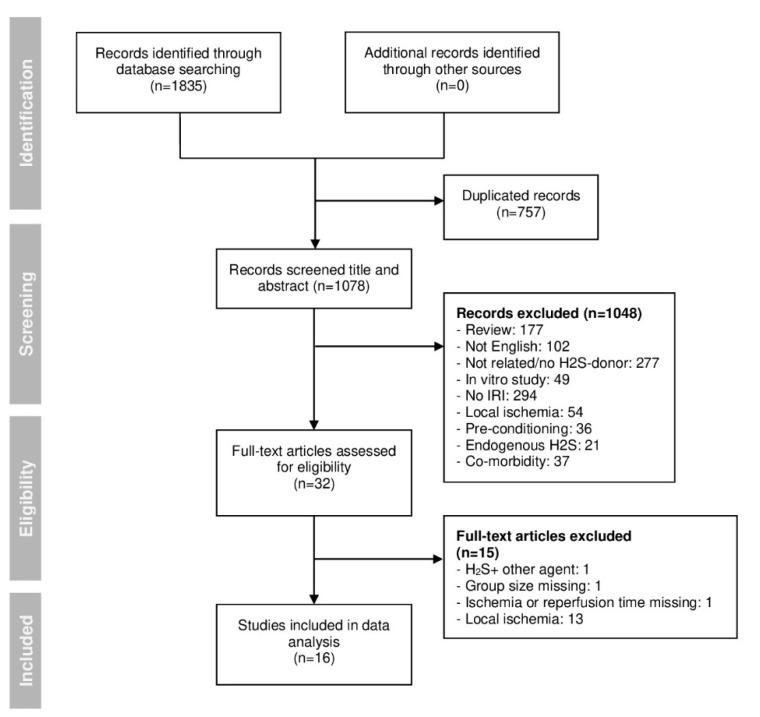
PRISMA flowchart of the search and selection process of the articles included in this review.

The initial electronic search identified 1835 studies. After eliminating duplicates, 1078 studies remained. The remaining studies were screened at the title and/or abstract level to check for relevancy to our study scope. Thirty-two studies were considered relevant and full-text reviewed. Finally, 16 studies were included in the analysis (Figure 1).

### 2.2. Main Characteristics

The characteristics of the included studies are summarized in Table 1. All studies were rodent models of IRI (rat *n* = 14, mouse *n* = 2). Sodium hydrosulfide (NaHS) was the most commonly used H_2_S donor (*n* = 12). Other used H_2_S donors were sodium thiosulfate (STS) (*n* = 3), GYY4137, DATS-MSN and sodium sulfate (Na_2_S) (all *n* = 1). The H_2_S donor was administered in the perfusate of Langendorff perfusion (*n* = 13) or in cold storage solution (*n* = 3). In Langendorff models, four studies used a postconditioning protocol whereby NaHS was applied in multiple rounds for 10–15 s (ischemic postconditioning) [16,17,18,19]. Evaluated variables relevant to our study scope are shown in Table 2. The definition of infarct size is the area of necrosis in the myocardial tissue. The method of choice to determine infarct size is by 2,3,5-triphenyltetrazolium (TCC) staining. This method has been shown to reliably identify a necrotic myocardium from a viable myocardium. The viable myocardium is stained red as the water-soluble compound TTC is converted by active mitochondrial dehydrogenases into an insoluble red precipitate. The extent of red staining correlates with the number of viable mitochondria and differentiates viable and nonviable tissue. The extent of the area of necrosis is quantified by computerized planimetry [20]. Data regarding inflammation were excluded for meta-analysis since only one study evaluated inflammation (not included in Table 2) [21]. In four of the included studies, multiple intervention groups had the same control group [17,18,21,22]. These additional intervention groups were excluded for meta-analyses. Exclusion was based on the least relevant H_2_S donor type, dosage and time of intervention.

### 2.3. Risk of Bias Assessment

**Figure 2:**
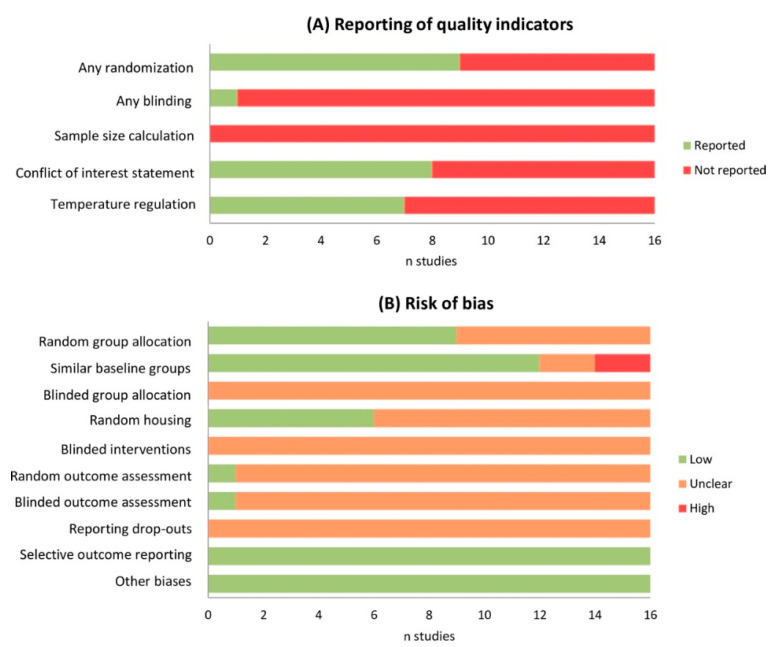
Quality and risk of bias assessment. Poor reporting of quality indicators (**A**) resulted in an unclear risk of bias for most types of bias (**B**). Selection bias = random group allocation, similar baseline groups and blinded group allocation. Performance bias = random housing and blinded intervention. Detection bias = random outcome assessment and blinded outcome assessment. Attrition bias = reporting of drop-outs. Reporting bias = selective outcome reporting.

The use of the SYRCLE risk of bias tool to assess the quality of animal studies indicated an unknown risk of bias for most studies in the majority of categories (Figure 2). The individual risk of bias scores can be found in Table S1. The funnel plot and results from Egger’s regression test showed significant publication bias (*p* < 0.001).

### 2.4. Meta-Analysis

#### 2.4.1. Infarct Size

**Figure 3:**
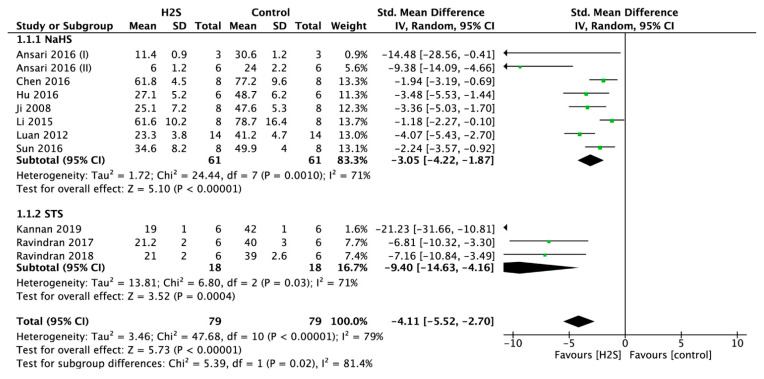
Forest plot of studies investigating the effect of H_2_S postconditioning on myocardial infarct size.

Infarct size measurements were performed in 11 studies. Postconditioning the heart using a H_2_S donor resulted in a significantly smaller infarct size (standardized mean difference (SMD) = −4.12, 95% confidence interval (CI) [−5.53–−2.71], *p* < 0.00001; Figure 3). This overall effect size was accompanied by a high degree of heterogeneity (I-square (I^2^) = 79%, *p* < 0.00001). In sensitivity analysis, the result remained consistent after excluding the studies one by one. In addition, subgroup analysis was performed with H_2_S donor groups NaHS or STS. In groups treated with STS, the infarct size was smaller when compared to groups treated with NaHS (SMD = −9.48, 95% CI [−14.74–−4.23] versus SMD = −3.05, 95% CI [−4.22–−1.87], *p* < 0.02; Figure 3).

#### 2.4.2. Cardiac Injury Markers

**Figure 4:**
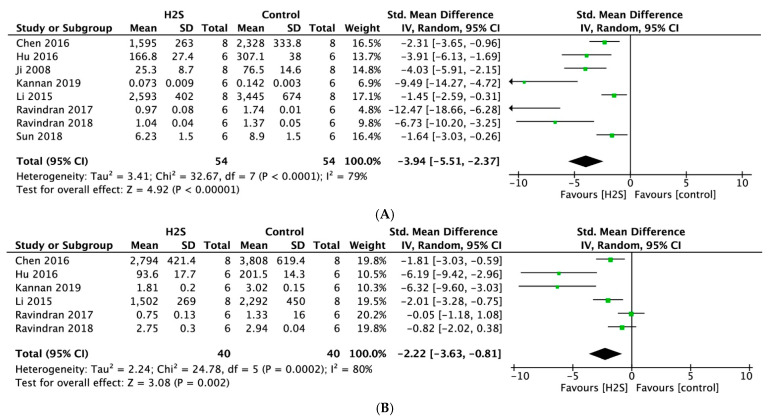
Forest plot of studies investigating the effect of H_2_S postconditioning on cardiac injury markers (**A**) CK and (**B**) LDH.

Meta-analysis of eight studies that reported on cardiac injury markers indicated that postconditioning with H_2_S resulted in significantly lower levels of creatine kinase (CK) compared with no treatment (SMD = −3.94, 95% CI [−5.51–−2.37], *p* < 0.00001; Figure 4A), with statistically significant heterogeneity (I^2^ = 79%, *p* < 0.0001). H_2_S treatment was also associated with significantly lower lactate dehydrogenase (LDH) levels measured in six studies (SMD = −2.22, 95% CI [−3.63–−0.81], *p* < 0.002; Figure 4B). Likewise, we observed a high degree of heterogeneity (I^2^ = 80%, *p* < 0.0002). For both outcomes, sensitivity analyses did not change the results.

#### 2.4.3. Oxidative Stress

**Figure 5:**
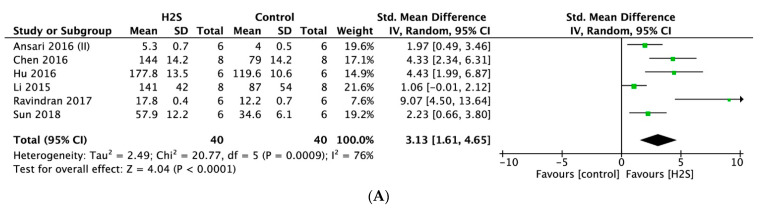

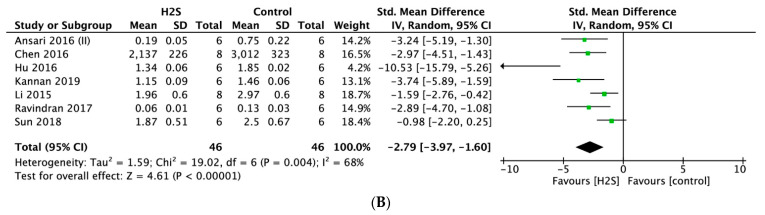
Forest plot of studies investigating the effect of H_2_S postconditioning on oxidative stress markers (**A**) SOD and (**B**) MDA.

Superoxide dismutase (SOD) was measured in six studies. Meta-analysis of these studies indicated that postconditioning with H_2_S was associated with significantly higher levels of SOD (SMD = 3.13, 95% CI [1.61–6.65], *p* = 0.0009; Figure 5A), with statistically significant heterogeneity (I^2^ = 86%, *p* < 0.00001). Meta-analysis of seven studies that reported on malondialdehyde (MDA) indicated that postconditioning with H_2_S was associated with significantly lower MDA levels compared to no treatment (SMD = −2.79, 95% CI [−3.97–−1.60], *p* < 0.00001; Figure 5B). This overall effect size was accompanied by a high degree of heterogeneity (I^2^ = 68%, *p* < 0.004). For both outcomes, sensitivity analyses did not change the results.

#### 2.4.4. Systolic Function

**Figure 6:**
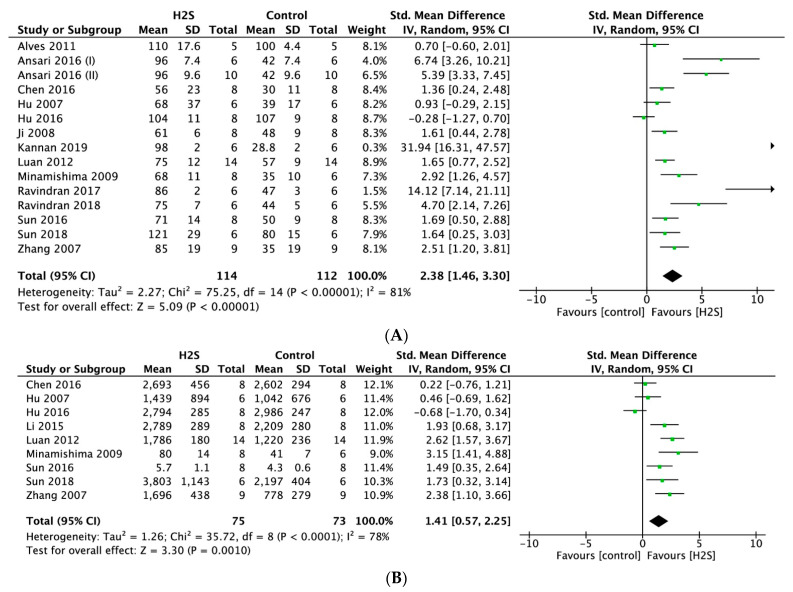
Forest plot of studies investigating the effect of H_2_S postconditioning on systolic function parameters (**A**) LVDP and (**B**) dP/dt max.

Meta-analysis of 15 studies indicated that postconditioning with H_2_S resulted in a significantly higher left ventricular (LV) developed pressure (LVDP) compared to no treatment (SMD = 2.38, 95% CI [1.46–3.30], *p* < 0.00001; Figure 6A), with statistically significant heterogeneity (I^2^ = 81%, *p* < 0.00001). Postconditioning the heart using a H_2_S donor resulted in a significantly higher maximum rate of LV pressure change (dP/dt_max_) (SMD = 1.41, 95% CI [0.57–2.25]; Figure 6B) compared with the control (*p* = 0.001, *n* = 9 comparisons). This overall effect size was accompanied by a high degree of heterogeneity (I^2^ = 78%, *p* < 0.0001). For both outcomes, sensitivity analyses did not change the results.

#### 2.4.5. Diastolic Function

**Figure 7:**
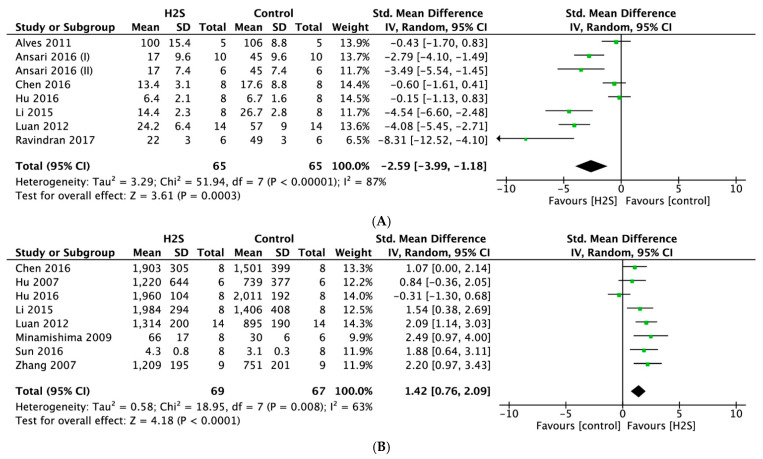
Forest plot of studies investigating the effect of H_2_S postconditioning on diastolic function parameters (**A**) LVEDP and (**B**) dP/dt min.

Postconditioning with a H_2_S donor resulted in a significantly lower LV end-diastolic pressure (LVEDP) (SMD = −2.59, 95% CI [−3.99–−1.18]; Figure 7A) compared with the control (*p* < 0.0003, *n* = 8 comparisons). This overall effect size was accompanied by a high degree of heterogeneity (I^2^ = 87%, *p* < 0.00001). In the treated group, H_2_S treatment also resulted in a significantly higher minimum rate of pressure change (dP/dt_min_) (SMD = 1.42, 95% CI [−0.76–2.09], *p* < 0.0001, *n* = 8 comparisons, Figure 7B). Likewise, a high degree of heterogeneity was observed (I^2^ = 63%, *p* = 0.008). For both outcomes, sensitivity analyses did not change the results.

#### 2.4.6. Heart Rate

**Figure 8:**
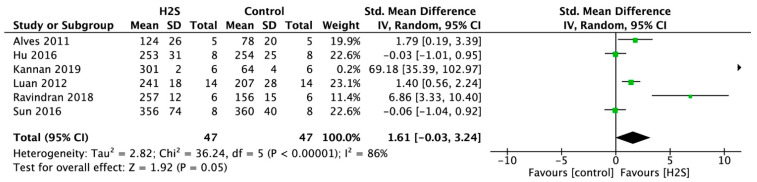
Forest plot of studies investigating the effect of H_2_S postconditioning on HR.

Postconditioning with a H_2_S donor resulted in a significantly higher heart rate (HR) (SMD = 1.61, 95% CI [−0.03–3.24]; Figure 8) compared with the control (*p* = 0.05, *n* = 6 comparisons). This overall effect size was accompanied by a high degree of heterogeneity (I^2^ = 86%, *p* < 0.00001). Sensitivity analyses did not change the results.

### 2.5. Meta-Regression Analysis

Meta-regression analyses found a significant association between the used H_2_S donor and CK (*p* = 0.008), and a trend towards a significant association between H_2_S donor and infarct size (*p* = 0.05) and SOD (*p* = 0.053). In these outcome variables, STS was more favorable than NaHS. Meta-regression analysis also found a significant association between route of administration and MDA (*p* = 0.04), with Langendorff being more favorable than CS. No significant association was identified for all other outcomes (LDH, SOD, LVDP, dP/dtmin, dP/dtmax, LVEDP and HR). Meta-regression of the postconditioning method (single bolus versus ischemic conditioning) showed no significant association with one of the outcomes as well.

## 3. Discussion

In the present systematic review and meta-analysis, we examined the existing experimental data on H_2_S postconditioning against global myocardial IRI in animal studies. We aimed at including all existing studies that used H_2_S treatment against IRI induced by low or absent coronary flow in the whole heart (global ischemia). Furthermore, we aimed to determine whether findings in this field are consistent, since clinical translation of H_2_S treatment for optimization of donor heart preservation could be of additional value. In all the 16 studies reviewed and in meta-analyses, we observed that H_2_S donors protect the heart against IRI. H_2_S postconditioning resulted in a significantly smaller infarct size, with greater effect in studies using STS as a H_2_S donor compared with studies using NaHS as a H_2_S donor. Furthermore, H_2_S postconditioning consistently resulted in significantly lower release of cardiac injury markers, lower markers of oxidative stress and higher cardiac function. Postconditioning with STS also showed significant advantageous effects in decreasing CK levels compared with NaHS. Nevertheless, the notable heterogeneity across studies must be highlighted. The infarct-limiting effects of H_2_S were also evaluated in local ischemia in vivo studies. Karwi et al. evaluated the effects of H_2_S pre- and postconditioning on myocardial infarction across in vivo preclinical studies using a comprehensive systematic review followed by meta-analysis [33]. This study showed significant infarct-sparing effects of H_2_S, which is in line with our results, emphasizing the infarct-limiting effects of H_2_S and potential clinical application in local and global myocardial protection against ischemia.

The most commonly used H_2_S donor in the reviewed papers is NaHS (*n* = 13), a fast H_2_S-releasing inorganic sulfide salt [34]. It was shown that NaHS can preserve cardiac function and reduce myocardial tissue damage in postconditioning studies. However, it is important to note that the cardioprotective effects of H_2_S depend on its physiological concentration. Subnormal tissue concentrations or low release rates of H_2_S have the most beneficial effect without toxicity [34]. However, at high concentrations or high release rates, H_2_S can induce toxicity and cell damage [35]. NaHS releases H_2_S instantaneously in aqueous solution, resulting in a high concentration of H_2_S within seconds and a short effective residence time in cardiac tissue [36]. To prevent the potential H_2_S toxicity, controllable and slow-releasing H_2_S donors have been designed and successfully demonstrated to have cardioprotective effects [21,36]. One of these controllable H_2_S sources is STS, a metabolite of the H_2_S detoxification process which can be adversely used to regenerate H_2_S. Apart from being a source of H_2_S, STS itself acts as a calcium chelator and antioxidant [23]. As mentioned before, STS treatment showed in this study more advantageous effects related to infarct size and levels of CK compared to NaHS treatment. STS has great potential due to both the H_2_S-related effects and the complementary effects of STS itself. Furthermore, additional anti-inflammatory and antioxidant effects of STS are related to its reaction with mitochondrial thiosulfate sulfurtransferase and its subsequent ROS scavenging effects [37]. In addition, pH-sensitive H_2_S donors have been developed including intramolecular cyclization-based donors (JK) and ammonium tetrathiomolybdate (ATTM) [38,39]. Since ischemic injury leads to reduced pH levels and acidosis, acid-promoted H_2_S release could be of significance in treating IRI. Both JK donors and ATTM have shown efficiency in reducing cellular damage and limiting infarct size in local ischemia models. In conclusion, slow-releasing and controllable H_2_S donors have more clinical potential than fast-releasing H_2_S donors.

In addition to paying attention to the H_2_S donor choice, the route of administration should be chosen carefully. In this study, subgroup analyses showed that H_2_S administered in Langendorff perfusate was more advantageous than H_2_S supplementation in CS solution with regard to lowered MDA levels. Further studies are needed to evaluate the effectiveness of H_2_S postconditioning in static CS versus H_2_S usage during reperfusion of the heart, for example, during Buckberg reperfusion [40].

The present study has some limitations. The literature search was restricted to the English language. We acknowledge that this restriction may have led to incomplete retrieval of relevant data. In addition, articles by the same research groups were included, assuming good adherence to scientific integrity, as in this way, valuable results were not overlooked. Lastly, we collected data on various markers of IRI without including time points for assessment of these markers. This might make the interpretation of the results difficult since more damage may occur over time, or, on the contrary, a longer treatment time may improve outcomes.

An important limitation we observed in the included studies is that none of the studies were performed in large animals. Although no single animal model perfectly recreates human pathophysiology, large animal models of cardiovascular research are more eligible for clinical translation than small animal models since determinants of myocardial work, e.g., HR, myocardial energy consumption and anatomy, are more similar between humans and large animals. Furthermore, the differences in the pharmacokinetics, distribution and metabolism of H_2_S between small and large animals may complicate the step to clinical translation [41]. Therefore, more large animal models for testing the efficacy and safety of H_2_S treatment against IRI are required before translating H_2_S therapy for organ preservation to the clinic. Another important aspect we observed in the animal studies is that none of the studies included female animals. Female hearts have an inherent cardioprotective advantage due to a lower inflammatory response in the ischemic myocardium compared to men [42]. Female rat hearts show significantly smaller infarct sizes than male hearts, indicating that there is a sex-specific difference in susceptibility to tissue damage induced by IRI [43]. Female animals or mixed gender studies need to be conducted to explore the sex differences in the susceptibility to IRI and efficiency of H_2_S treatment against IRI. Furthermore, when looking at study models, all included studies performed beating heart procurements, and none of the studies applied H_2_S postconditioning in a DCD procedure. Therefore, further studies need to be conducted to explore the possibilities to preserve DCD heart function with H_2_S, in order to enlarge the donor pool for transplantation with DCD hearts.

Long-term effects of H_2_S treatment were described in only one of the sixteen papers we reviewed (8-week follow-up time) [21]. Mostly, cardioprotective effects by H_2_S were investigated after a single dose shortly before ischemia or after reperfusion. As stated previously, transplantation of the treated heart would require long-term evaluation of the cardioprotective effects on the heart. Further (transplantation) studies are needed to determine time-dependent effects of H_2_S treatment against IRI and evaluate possible beneficial and detrimental effects on all organs, especially in larger animal models since long-term effects of H_2_S postconditioning against IRI in large animals are not known yet.

## 4. Materials and Methods

This study was conducted according to the Preferred Reporting Items for Systematic Reviews and Meta-Analysis (PRISMA) guidelines [44].

### 4.1. Search Strategy

A systematic electronic literature search was performed using Medline, Cinahl and Embase databases, from inception to 25 August 2020. We used combinations of search terms and keywords including “heart”, “organ transplantation”, “organ preservation”, “hydrogen sulfide” and “reperfusion injury”. Full search terms can be found in Table S2. We performed hand searching of the reference list of selected articles from the electronic searches to ensure inclusion of any studies not found by the primary search.

### 4.2. Eligibility Criteria

Eligibility criteria were developed in accordance with the PICOS approach [45]. Studies were included if they met the following eligibility criteria: (1) original article examining the effects of postconditioning the heart with H_2_S to limit IRI; (2) in situ or ex situ study; (3) myocardial ischemia was induced by complete cessation of coronary flow (global ischemia model); (4) one or more of the following outcome variables were included in the study: myocardial infarct size, cardiac injury markers, inflammation, oxidative stress or cardiac function. Studies without a documented ischemia and reperfusion time, type of exogenous H_2_S donor, H_2_S donor dosage, timing and route of administration were excluded. Studies were also excluded if the H_2_S donor was administered in combination with another pharmacological treatment, and studies involving animal models with induced non-coronary/non-myocardial diseases at baseline were also excluded.

### 4.3. Data Extraction

Data extraction was performed by one reviewer. If inclusion of articles was questionable, a second and a third reviewer reviewed these particular articles. Publications were retrieved from the electronic literature databases and checked for duplication. Initially, the references were reviewed based on the titles and abstracts. Then, relevant articles were fully read and assessed. Study characteristics were divided into first author, publication year, species, gender, number of included animals, study type (in or ex situ), H_2_S donor, dosage, time of intervention and route of administration of the H_2_S donor. The primary outcome variable was myocardial infarct size. Secondary outcome variables were divided into cardiac injury markers, inflammation, oxidative stress and functional parameters.

### 4.4. Risk of Bias Assessment

Risk of bias was assessed using SYRCLE’s RoB tool [46]. This tool, based on the Cochrane Collaboration RoB Tool, aims to assess methodological quality and has been adapted to aspects of bias that play a role in animal experiments, including (1) selection bias, (2) performance bias, (3) detection bias, (4) attrition bias and (5) reporting bias.

### 4.5. Statistical Analysis

All outcome measures were treated as continuous data. Data were presented as SMD with 95% CI. Every outcome evaluated in at least five studies was analyzed. The random effects model was used for pooling results. The H_2_S-treated group was compared with the control group (no treatment/placebo) by use of the weighted mean difference (WMD). Heterogeneity was quantified using the I^2^ statistics test. An I^2^ higher than 50% was considered indicative of significant heterogeneity. Forest plots were created to summarize the meta-analysis study results. Meta-regression analyses were performed to examine an association between type of H_2_S donor, postconditioning method (single bolus or ischemic conditioning) and route of administration in all outcome variables. Publication bias was assessed by inspection of funnel plots and quantified by Egger’s test. A *p*-value < 0.05 was considered statistically significant. Statistical analyses and figures were performed and composed with Review Manager (RevMan 5.3.5 Copenhagen, Denmark: The Nordic Cochrane Centre, The Cochrane Collaboration, 2014) and Stata (StataCorp, College, TX, USA).

## 5. Conclusions

This systematic review provides an overview of the cardioprotective effects of H_2_S postconditioning against IRI in the setting of global ischemia. The included studies showed robust beneficial effects of H_2_S postconditioning with regard to infarct size, cardiac injury markers, oxidative stress and cardiac function. Postconditioning with slow-releasing H_2_S donors might offer a valuable opportunity for novel therapies within cardiac preservation for transplantation. Before clinical implication, studies evaluating the (long-term) effects of H_2_S treatment in large and female/mixed animal studies are warranted.

## Figures and Tables

**Table 1:** Main characteristics of included studies. CS = cold storage; m = male; n/g = not given; Na_2_S = sodium sulfide; NaHS = sodium hydrosulfide; R = reperfusion; STS = sodium thiosulfate. The main characteristics included: (1) ID, (2) Author, (3) Publication year, (4) Species (gender), (5) Number of animals (experimental/control), (6) Study type, (7) H_2_S donor, (8) Dosage, (9) Time of intervention (during CS/at time of reperfusion), (10) Route of administration, (11) Warm ischemic time (min), (12) Cold ischemic time (h) and (13) Reperfusion time (min).

1	2	3	4	5	6	7	8	9	10	11	12	13
1	Kannan et al. [23]	2019	Rat (m)	6/6	Ex vivo	STS	1 µM	R	Langendorff	30	0	60
2	Sun et al. [21] (A)	2018	Rat (m)	6/6	Transplant	NaHS	25 µM	CS	CS solution	0	4	60
	Sun et al. [21] (B)	2018	Rat (m)	6/6	Transplant	GYY4137	5 µg/mL	CS	CS solution	0	4	60
	Sun et al. [21] (C)	2018	Rat (m)	6/6	Transplant	DATS-MSN	3 µg/mL	CS	CS solution	0	4	60
3	Ravindran et al. [24]	2018	Rat (n/g)	6/6	Ex vivo	STS	1 µM	R	Langendorff	30	0	60
4	Ansari et al. (I) [25]	2016	Rat (m)	3/3	Ex vivo	NaHS	20 µM	R	Langendorff	30	0	75
5	Ravindran et al. [26]	2017	Rat (m)	6/6	Ex vivo	STS	1 µM	R	Langendorff	30	0	60
6	Ansari et al. (II) [27]	2016	Rat (m)	6/6	Ex vivo	NaHS	20 µM	R	Langendorff	30	0	60
7	Hu et al. [16]	2016	Rat (m)	6/6	Ex vivo	NaHS	10 µM	R, 4 × 15 s	Langendorff	30	0	60
8	Chen et al. [17] (A)	2016	Rat (m)	8/8	Ex vivo	NaHS	10 μM	R	Langendorff	40	0	60
	Chen et al. [17] (B)	2016	Rat (m)	8/8	Ex vivo	NaHS	10 μM	R, 6 × 10 s	Langendorff	40	0	60
9	Zhang et al. [28]	2007	Rat (m)	9/9	Ex vivo	NaHS	40 µM	R	Langendorff	30	0	30
10	Sun et al. [29]	2016	Mouse (m)	8/8	Ex vivo	NaHS	100 µM	R	Langendorff	20	0	90
11	Ji et al. [22] (A)	2008	Rat (m)	8/8	Ex vivo	NaHS	10 µM	R	Langendorff	30	0	90
	Ji et al. [22] (B)	2008	Rat (m)	8/8	Ex vivo	NaHS	0.1 µM	R	Langendorff	30	0	90
	Ji et al. [22] (C)	2008	Rat (m)	8/8	Ex vivo	NaHS	1 µM	R	Langendorff	30	0	90
	Ji et al. [22] (D)	2008	Rat (m)	8/8	Ex vivo	NaHS	100 µM	R	Langendorff	30	0	90
12	Luan et al. [19}	2012	Rat (m)	14/14	Ex vivo	NaHS	10 µM	R, 4 × 15 s	Langendorff	30	0	90
13	Hu et al. [30]	2007	Rat (n/g)	6/6	Ex vivo	NaHS	1 µM	CS	CS solution	0	6	30
14	Alves et al. [31]	2010	Rat (m)	5/5	Ex vivo	NaHS	100 µM	CS	CS solution	0	4	30
15	Li et al. [18] (A)	2015	Rat (m)	8/8	Ex vivo	NaHS	10 µM	R	Langendorff	40	0	60
	Li et al. [18] (B)	2015	Rat (m)	8/8	Ex vivo	NaHS	10 µM	R, 5 × 10 s	Langendorff	40	0	60
16	Minamishima et al. [32]	2009	Mouse (m)	8/6	Ex vivo	Na_2_S	10 µM	R	Langendorff	20	0	60

**Table 2:** Evaluated variables in included studies. * timing of evaluation: 30 min WIT, 15 min R. AI, apoptosis index; BAX, Bcl-2-associated X protein; Bcl-2, B-cell lymphoma 2 protein; C3, caspase-3; C9, caspase-9; CAT, catalase; CK, creatine kinase; CI, cardiac injury; dP/dt_max_, maximal pressure rise; dP/dt_min_, minimal pressure rise; EF, ejection fraction; FR, flow rate; GPx, glutathione peroxidase; GR, glutathione reductase; GSH, glutathione; HR, heart rate; LDH, lactate dehydrogenase; LVDP, left ventricular develop pressure; LVEDP, left ventricular end-diastolic pressure; MDA, malondialdehyde; PGC-1α, peroxisome proliferator-activated receptor gamma coactivator 1-alpha; RPP, rate pressure product; SOD, superoxide dismutase; STAT3, signal transducer and activator of transcription 3.

ID	Author	Infarct Size	CI Markers	Apoptosis Markers	Oxidative Stress Markers	Cardiac Function	Timing of Evaluation
1	Kannan et al. [23]	Yes	CK, LDH		GSH, SOD, CAT, GPx, GR	LVDP, HR, RPP	90 min (30 min WIT, 60 min R)
2	Sun et al. [21]	No	CK, cTnI	C3, BAX, AI	GSH, SOD, CAT, MDA	LVDP, dP/dt_max_, EF, arrhythmia	270 min (240 min CIT, 30 min R)
3	Ravindran et al. [24]	Yes	CK, LDH		SOD, CAT, GPx, GR, PGC-1α	LVDP, HR	90 min (30 min WIT, 60 min R)
4	Ansari et al. [25]	Yes	CK, LDH	C3		LVDP, LVEDP, RPP	105 min (30 min WIT, 75 min R)
5	Ravindran et al. [26]	Yes	CK, LDH		GSH, SOD, CAT, GR, GPx, MDA	LVDP, LVEDP, dP/dt_max_, RPP	90 min (30 min WIT, 60 min R)
6	Ansari et al. [27]	Yes	CK, LDH	C3	GSH, CAT, GR, GPx, MDA	LVDP, LVEDP, RPP	90 min (30 min WIT, 60 min R)
7	Hu et al. [16]	Yes	CK, LDH		SOD, MDA, PGC-1α	LVDP, LVEDP, LVDP, LVEDP, dP/dt_max_, RPP	90 min (30 min WIT, 60 min R)
8	Chen et al. [17]	Yes	CK, LDH	Bcl-2, AI	SOD, MDA	LVDP, LVEDP, LVDP, LVEDP, dP/dt_max_	100 min (40 min WIT, 60 min R)
9	Zhang et al. [28]	No				LVDP, dP/dt_max_, arrhythmia	60 min (30 min WIT, 30 min R)
10	Sun et al. [29]	Yes				LVDP, LVEDP, dP/dt_max_ and dP/dt_min_,HR, RPP, FR	110 min (20 min WIT, 90 min R)
11	Ji et al. [22]	Yes	CK *			LVDP, HR	120 min (30 min WIT, 90 min R)
12	Luan et al. [19]	Yes		Bcl-2, BAX, STAT3, AI		LVDP, LVEDP, dP/dt_max_, HR	120 min (30 min WIT, 90 min R)
13	Hu et al. [30]	No		AI		LVDP, dP/dt_max_, dP/dt_min_	390 min (360 min CIT, 30 min R)
14	Alves et al. [31]	No		Bcl-2		LVDP, LVEDP, HR	270 min (240 min CIT, 30 min R)
15	Li et al. [18]	Yes	CK, LDH	C3, C9, Bcl-2, AI	SOD, MDA	LVDP, LVEDP, dP/dt_max_. dP/dt_min_	100 min (40 min WIT, 60 min R)
16	Minamishima et al. [32]	No				LVDP, dP/dt_max_, dP/dt_min_, RPP, FR	80 min (20 min, WIT 60 min R)

## Data Availability

No new data were created or analyzed in this study. Data sharing is not applicable to this article.

## Supplementary Materials

The following are available online at https://www.mdpi.com/article/10.3390/ijms22115737/s1.
