# Supplementary figures and images for: Donor Heart Preservation with Hydrogen Sulfide: A Systematic Review and Meta-Analysis
Source: Int J Mol Sci. 2021 May 27;22(11):5737. doi: 10.3390/ijms22115737 (PMC8198118; doi:10.3390/ijms22115737)

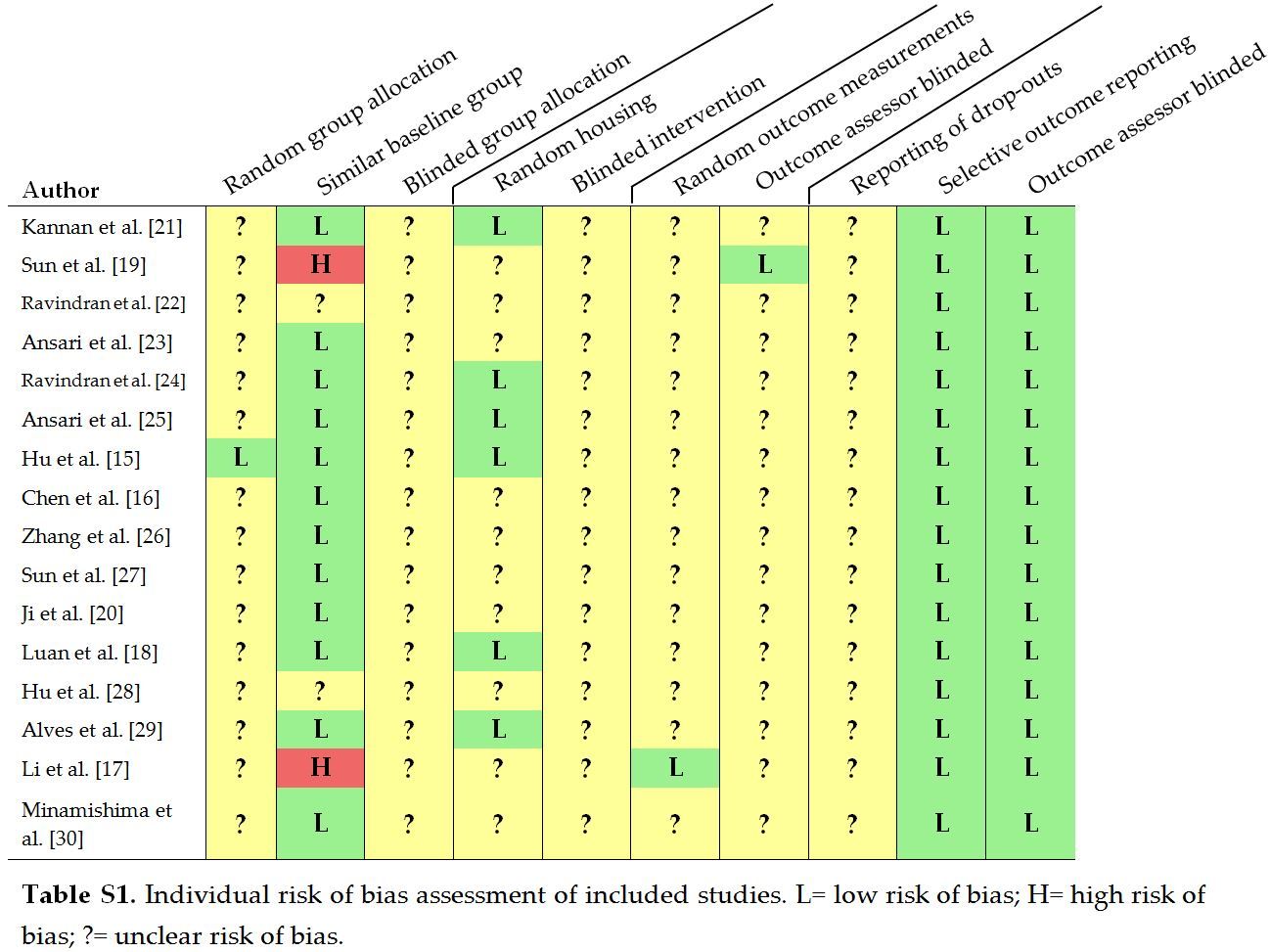

Supplement: Supplementary file 1 [file ijms-22-05737-s001.zip › Table S1. Individual risk of bias assessment of included studies.jpg]

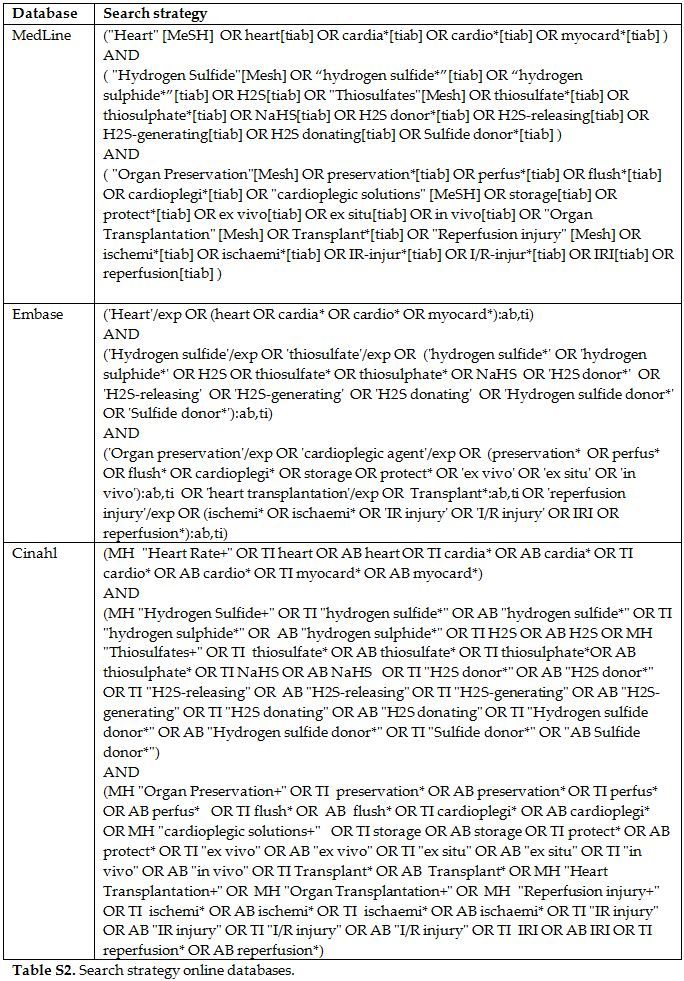

Supplement: Supplementary file 1 [file ijms-22-05737-s001.zip › Table S2. Search strategy online databases.jpg]
